# A Case of Persistent *Bacillus cereus* Bacteremia Responding to a Combination of Vancomycin and Gentamicin

**DOI:** 10.1155/2022/8725102

**Published:** 2022-03-12

**Authors:** Hiroshi Sasano, Toshihiro Yoshizawa, Mai Suzuki, Yukiko Fukui, Ryutarou Arakawa, Naoto Tamura, Toshio Naito

**Affiliations:** ^1^Department of Pharmacy, Juntendo University Hospital, Tokyo, Japan; ^2^Department of General Medicine, Juntendo University Faculty of Medicine, Tokyo, Japan; ^3^Department of Internal Medicine and Rheumatology, Juntendo University School of Medicine, Tokyo, Japan

## Abstract

A 56-year-old woman with a history of connective tissue disease developed fever, and *Bacillus cereus* (*B. cereus*) was detected in blood cultures. Therefore, treatment with vancomycin (VCM) was initiated. Since her blood cultures persistently detected *B. cereus* despite peripheral intravenous catheter replacement and VCM treatment, concomitant treatment with gentamicin (GM) was started. Blood cultures then became negative. Persistent *B. cereus* bacteremia responded to combination therapy with VCM and GM. This combination therapy may increase the risk of developing renal dysfunction, but the risk can be mitigated by appropriate therapeutic drug monitoring (TDM) and dose adjustments to achieve successful treatment.

## 1. Introduction

*Bacillus cereus* (*B. cereus*), a spore-forming, aerobic, Gram-positive bacilli, is an environmental microorganism found widely in nature, including soil and dust. In daily clinical practice, it is often considered a contaminant, even if detected in blood cultures. However, in neonates and immunocompromised patients, *B. cereus* has been reported to cause serious bloodstream infections [1–3]. This was a case report of persistent bacteremia with *B. cereus* repeatedly detected in multiple blood cultures, successfully treated with a combination of vancomycin (VCM) and gentamicin (GM) with therapeutic drug monitoring (TDM) and appropriate dose adjustments, without adverse events such as nephrotoxicity.

## 2. Case

A 56-year-old woman developed rectal pain and blood-tinged stools starting at the end of December 2019 and presented to the emergency department as an outpatient on January 3. Plained computed tomography (CT) showed rectal fecal impaction, which was evacuated following an enema. The patient went home, but she presented again the next day, complaining of poor dietary intake and poor general condition, and she was subsequently admitted for evaluation and care.

Her past medical history included polymyositis, Sjögren's syndrome, scleroderma, pulmonary hypertension, interstitial pneumonia, and dermatological ulcers. Her past medical history included an allergy to levofloxacin (with eosinophilia).

On physical examination, her vital signs were as follows: temperature, 38.1°C; pulse, 100/min; respiratory rate, 14/min; blood pressure, 102/82 mmHg; and SpO_2_, 98% (on O_2_ at 3 L). There was no evidence of anemia or jaundice. Her breath sounds were clear, and her heart sounds were regular, with no murmurs. Her abdomen was flat and soft, her bowel sounds were normal, and spontaneous pain was absent. Perianal skin peeling and local tenderness were observed.

On January 4, the patient was given peripheral intravenous infusions of nutrient preparation and soft-food products for nutritional improvement. On January 9, the patient passed bloody stool and experienced intense rectal pain, which prevented endoscopy; thus, she was managed with symptomatic treatment and watchful waiting. On January 11, the oral nutrient intake was temporarily suspended. With no bloody stool noted subsequently, oral food intake resumed on January 14, advancing from a fluid diet to solid food. On January 16, the patient had a fever of 38°C but no marked changes in her vital signs or general condition. Despite acetaminophen treatment, blood work on the following day (January 17) confirmed a high inflammatory response. Blood, urine, and sputum specimens were cultured for suspected infections. The absence of abdominal pain or changes in respiratory condition gave no suggestions for a potential focus. Nevertheless, with the chest X-ray findings of worsened lung field shadows, four times daily (every 6 hours) treatment with tazobactam/piperacillin 4.5 g was started. A blood culture report on January 18 showed Gram-positive bacilli. Considering the involvement of microbes such as *B. cereus*, treatment with VCM was initiated. The final blood culture report identified the microbes to be *B. cereus* in two of two sets. When the serum trough concentration of VCM on the same day was high, at 21.7 *μ*g/mL, the VCM dose was reduced from 2 to 1 g/day. To confirm resolution, two sets of blood cultures were collected again on January 20 and 22, but *B. cereus* was again detected in two of two sets from both days. With the serum trough concentration of VCM at 11.8 *μ*g/mL on January 23, the VCM dose was increased to 1.5 g/day. *B. cereus* was detected in one of two sets of blood cultures collected on January 25. The serum trough concentration of VCM on January 27 was 15.5 *μ*g/mL. Moreover, contrast-enhanced CT on the same day revealed no signs suggestive of abscess formation. *B. cereus* was again detected in one of two sets of blood cultures collected on January 28.

**Figure 1:**
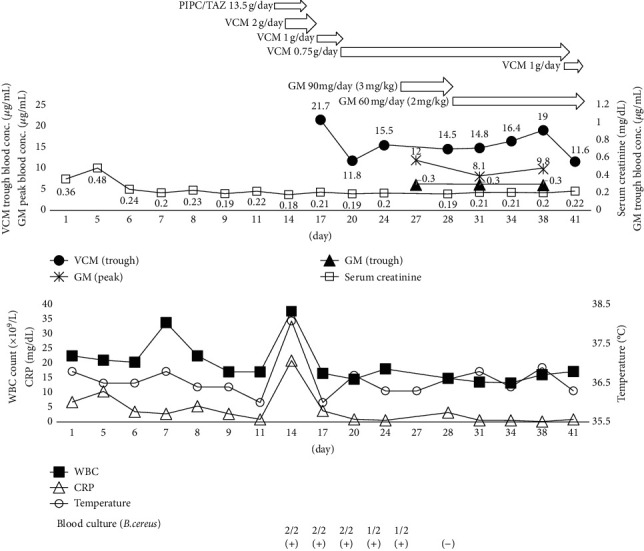
The patient's clinical course. PIPC/TAZ: piperacillin/tazobactam, VCM: vancomycin, and GM: gentamicin.

Given the persistently positive culture results, treatment with 90 mg GM (3 mg/kg) was administered on January 29. The serum concentration of GM on January 30 was 12.0 *μ*g/mL at the peak and 0.3 *μ*g/mL at the trough, within normal. Nevertheless, given that its concomitant use with VCM is associated with potential impairment of renal function, the GM dose was reduced to 60 mg (2 mg/kg) per day. Thereafter, the VCM and GM doses were adjusted as appropriate depending on serum concentrations. Blood cultures were repeated on January 31, and the final report on February 5 confirmed negative blood cultures. With January 31 as the initial date, the patient completed a total of 14 subsequent days of treatment with a combination of VCM and GM (Figure 1).

## 3. Discussion

Most *Bacillus* infections in humans are caused by *B.* cereus [4, 5]. Infections by *B. cereus* may present as endocarditis, bacteremia, meningitis, pneumonia, skin/soft tissue infection, and food poisoning [6], and they are seen in at-risk populations such as neonates and immunocompromised patients [1–3]. *B. cereus* detected in blood cultures is often attributed to contamination during blood sampling but may include isolates from a single set of blood culture bottles and repeat isolates from multiple blood cultures [7, 8].

*B. cereus* produces *β*-lactamase and is generally resistant to *β*-lactam antibiotics, including third-generation cephalosporins, thus making VCM and GM the drugs of choice [6, 9, 10]. In addition to antibiotic treatment, intravascular catheter removal is considered effective [11]. In the present case, treatment with VCM was started based on a blood culture result showing Gram-positive bacilli. Therapeutic drug monitoring (TDM) of serum concentrations is warranted for VCM, with the effective serum trough concentration reported to be in the range of 10 to 20 *μ*g/mL [12]. Several case reports and literature reviews have demonstrated the successful treatment of *Bacillus* spp. infection with VCM [13–15]. However, there was no mention of target concentration levels during treatment. Therefore, in the present case, TDM was performed to ensure that the trough concentrations were maintained between 10 and 20 *μ*g/mL. Furthermore, instead of central venous catheters, only peripheral intravenous catheters were used to administer infusions and the catheters were replaced as appropriate. Despite such efforts, *B. cereus* was persistently detected in the blood cultures.

There have been several case reports of synergistic antimicrobial effects from the concomitant use of VCM and GM in treating severe *B. cereus* infections [16–20]. GM was added in the present case, resulting in the subsequent elimination of *B. cereus* in blood cultures.

TDM is warranted for GM and other target concentration levels, similar to VCM. Eccicacy and nephrotoxicity are reportedly related to the peak and trough concentrations, respectively. Cases of successful treatment with vancomycin and aminoglycoside antibacterial agents have been reported [16–20]. However, no specific target peak or trough concentrations are available for treating infections with Gram-positive bacilli, such as *B. cereus*. In the present case, doses were adjusted as appropriate by TDM using the guidelines for management of infective endocarditis [21, 22]. There have been reports of concomitant VCM-induced increases in renal impairment risk [23] and significant decrease in aminoglycoside-induced nephrotoxicity with a single-dose versus a multiple-dose regimen [24]. The adjustments allowed the patient to complete the treatment appropriately without developing renal complications.

## 4. Conclusions

The combination therapy of VCM and GM was effective in treating persistent *B. cereus* bacteremia. Although increase in nephrotoxicity with the concomitant administration of VCM and aminoglycoside antibacterial agents is known, performing TDM and appropriate dose selection can prevent adverse events and make successful treatment possible for Gram-positive rod bacteremia.
